# Descemet membrane endothelial keratoplasty in a patient with iris-fixated intraocular lens and prior radial keratotomy: a case report

**DOI:** 10.1186/s12886-021-02103-1

**Published:** 2021-09-20

**Authors:** Anvesh Annadanam, Timothy Soeken, Manjool Shah, Nambi Nallasamy

**Affiliations:** grid.214458.e 0000000086837370 W. K. Kellogg Eye Center, University of Michigan, 1000 Wall St, MI 48105 Ann Arbor, USA

**Keywords:** DMEK, RK, Iris-fixated IOL, Case report

## Abstract

**Background:**

Anterior segment surgeries such as cataract surgery, intraocular lens (IOL) repositioning, and radial keratotomy (RK) may hasten endothelial dysfunction, particularly in the context of pre-existing Fuchs dystrophy, necessitating future corneal transplantation.

**Case presentation:**

A 68-year-old woman with a history of RK with associated irregular astigmatism in both eyes and iris-fixated intraocular lens (IF-IOL) in the left eye presented with six months of decreased vision in the left eye. She was found to have Fuchs dystrophy and underwent DMEK surgery. She had an uncomplicated postoperative course, with uncorrected visual acuity improving to 20/20 three months after surgery.

**Conclusion:**

To our knowledge, this is the first reported case of a highly successful DMEK surgery in a patient with prior RK and IF-IOL.

**Supplementary Information:**

The online version contains supplementary material available at 10.1186/s12886-021-02103-1.

## Background

Descemet membrane endothelial keratoplasty (DMEK) is a well-established method of corneal transplantation for the treatment of endothelial dysfunction in Fuchs dystrophy [1]. Radial keratotomy (RK) is a method of refractive surgery historically used to correct myopia, involving the creation of radial incisions to flatten the central cornea. Popular in the 1980 s, RK is now largely disfavored due to its numerous complications [2, 3], including irregular astigmatism. Fuchs dystrophy in patients with a history of RK has previously been managed with either Descemet stripping automated endothelial keratoplasty (DSAEK) or penetrating keratoplasty (PK) [4]. More recently, DMEK had been successfully performed in this population [5, 6].

DMEK can be a challenging procedure to perform on post-vitrectomy eyes and those with potentially unstable intraocular lenses (IOL), such as scleral-fixated IOLs and iris-fixated IOLs (IF-IOLs). Injection of air or sulfur hexafluoride (SF_6_) gas into the anterior chamber after DMEK may potentially dislocate these precarious lenses. However, cases of successful DMEK surgery in patients with scleral-fixated and IF-IOLs have recently been reported [7].

We present a challenging case of a woman who underwent DMEK for corneal endothelial decompensation in the setting of Fuchs dystrophy, with a history of both RK and IF-IOL, as well as prior anterior vitrectomy. To our knowledge, this represents the first report of successful DMEK in a patient with both RK and IF-IOL.

## Case presentation

**Fig. 1:**
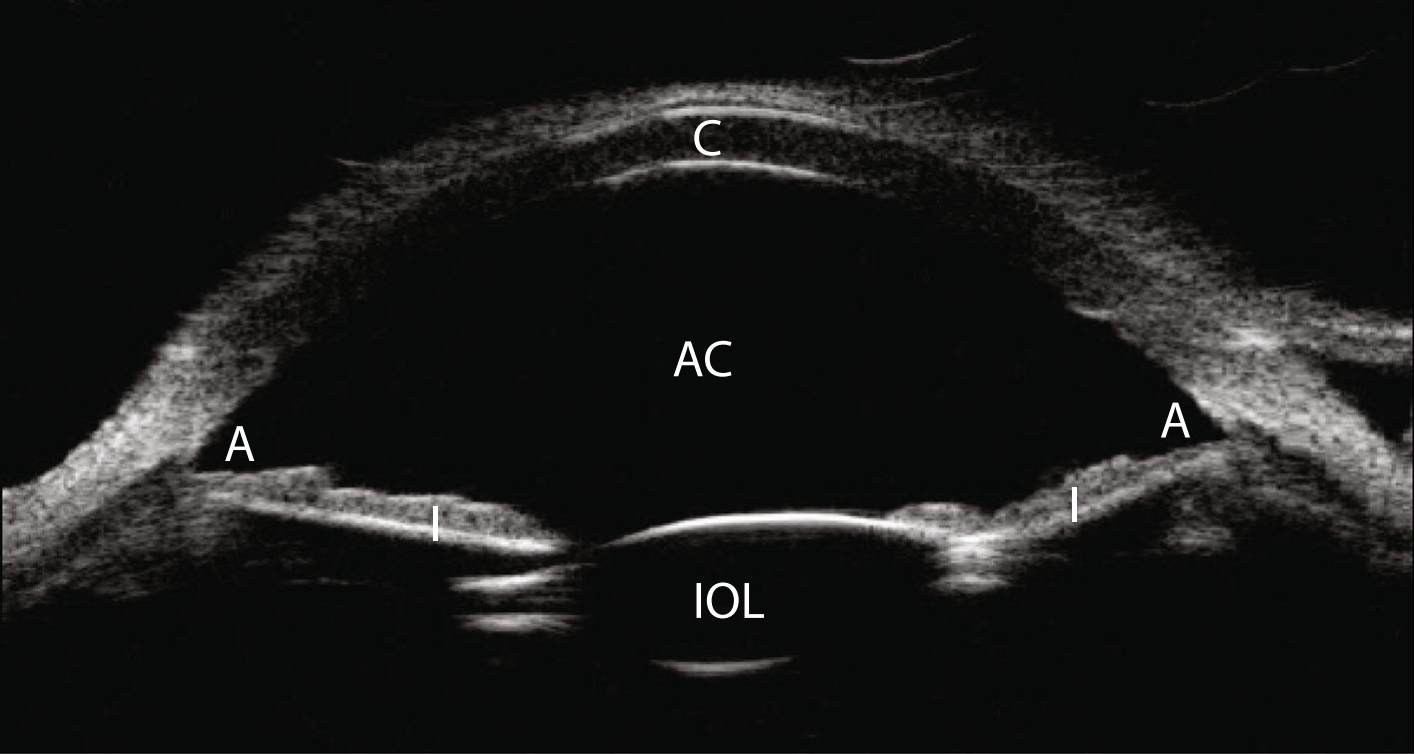
Horizontal axial view of ultrasound biomicroscopy study showing the relationship of anterior segment structures prior to endothelial keratoplasty. C – cornea, AC – anterior chamber, A – iridocorneal angle, I – iris, IOL – iris-fixated intraocular lens

A 68-year-old woman was referred to a tertiary eye center for evaluation of Fuchs dystrophy of the left eye. She had reported a 6-month history of decreasing visual acuity (VA), with the left eye worse than the right eye. Her past ocular history includes anterior RK in both eyes 25 years prior to presentation. She had also had cataract extraction in both eyes with in-the-bag IOL placement five years prior to presentation. Following cataract surgery, the patient experienced significant negative dysphotopsias in the left eye and underwent IOL exchange four months after cataract surgery with placement of the new IOL in the ciliary sulcus. Three years later, her sulcus IOL was found to be inferiorly displaced and was repositioned into the capsular bag with placement of a capsular tension segment. A year and a half later, she continued to experience negative dysphotopsias. She underwent repeat IOL exchange with iris-suturing of a Bausch and Lomb 3-piece LI61AO 23.0 diopter lens and anterior vitrectomy, with the intention of addressing negative dysphotopsias by decreasing the depth of the posterior chamber (Fig. 1). Two months following this surgery, her best corrected visual acuity (BCVA) was 20/40 in the right eye (OD) and 20/70 in the left eye (OS), but she continued to experience negative dysphotopsias OS. Optical coherence tomography (OCT) of the left macula showed epiretinal membrane (ERM), and she was referred to a local retina specialist for evaluation. At that visit, she was found to have corneal edema of the left eye, diagnosed with Fuchs corneal dystrophy, and referred to our service for assessment. The ERM was deemed to be non-surgical at the time.

**Fig. 2:**
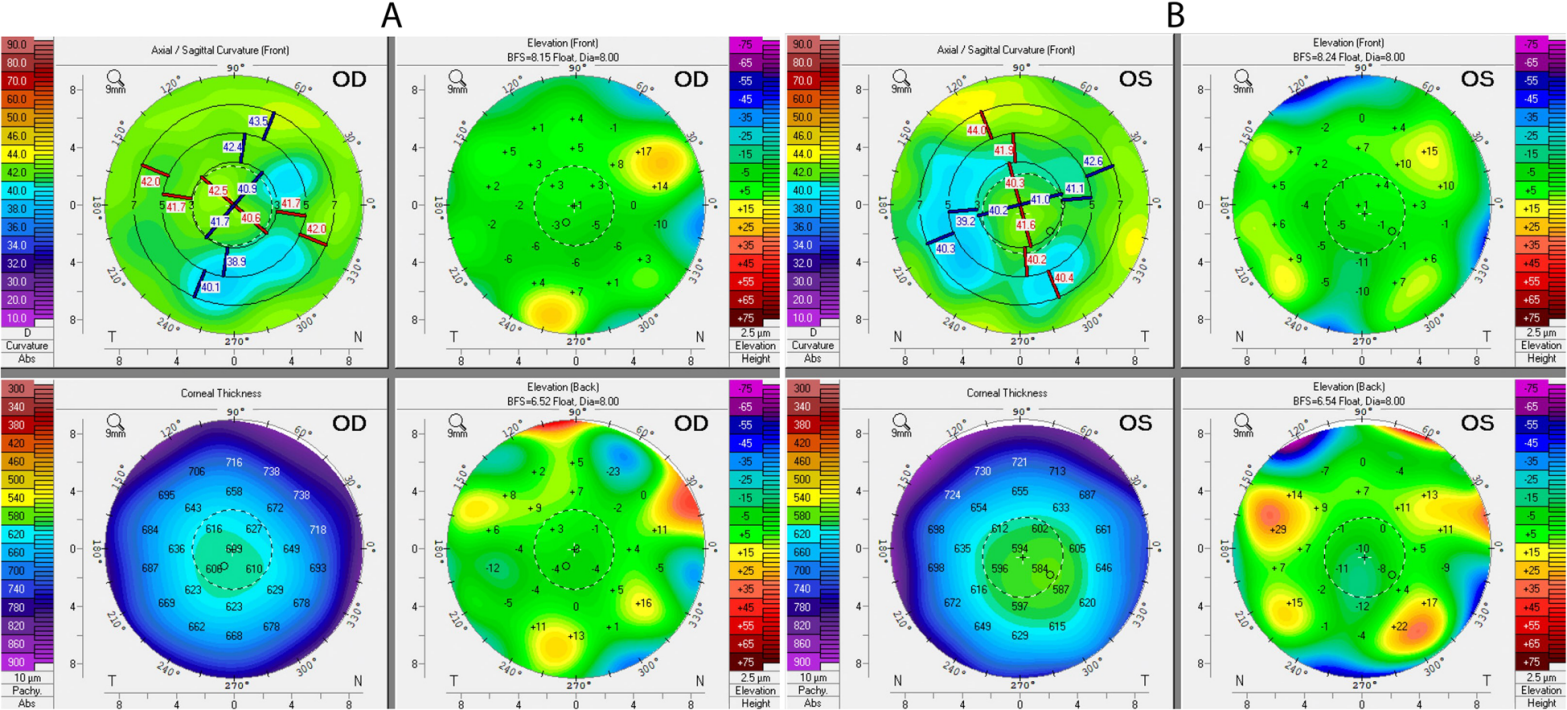
Pentacam of left cornea. (**A**) Preoperative scan showing irregular astigmatism, central corneal flattening, and diffuse corneal edema. (**B**) Postoperative scan showing improved central corneal architecture, decreased astigmatism, and markedly decreased edema

**Fig. 3:**
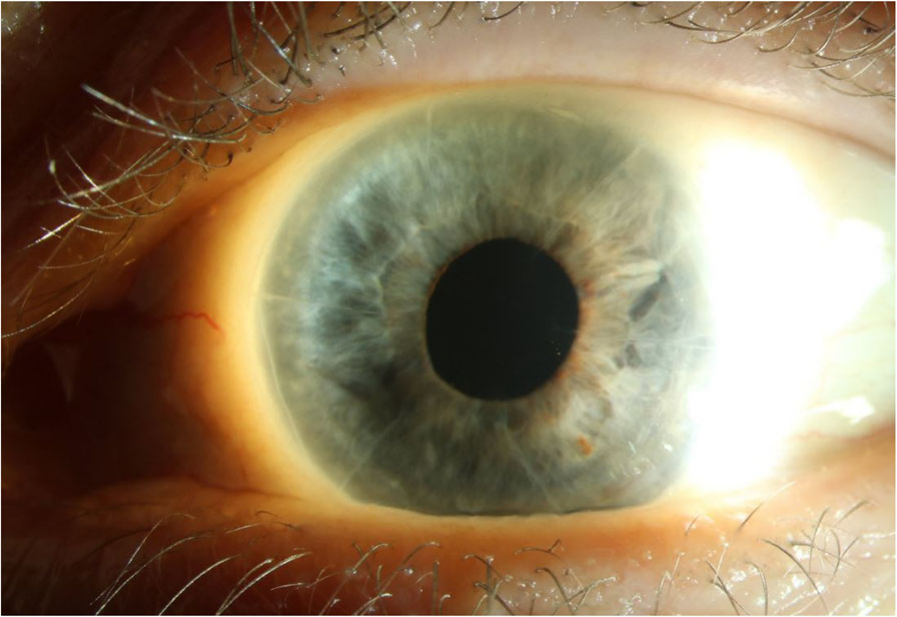
Postoperative slit lamp photo of the left eye showing a clear cornea

On our evaluation, she was found to have a BCVA of 20/30 OD and 20/80 OS, corneal pachymetry of 579 μm OD and 621 μm OS, no visible edema OD and 1 + stromal edema OS, and 2 + guttae OD and 3 + pigmented guttae OS. Six RK incisions were identified in both eyes (OU). Her IF-IOL was well-positioned OS, with haptics fixated at two points with 10 − 0 Prolene suture, and slight ovalization of the pupil. Preoperative corneal Scheimpflug imaging demonstrated irregular astigmatism OU (Fig. 2). An inferior peripheral laser iridotomy was performed and the patient underwent an uneventful DMEK surgery OS. The donor DMEK tissue (8.0mm diameter) was obtained pre-loaded from the eye bank. A Dutch Ophthalmic Research Center (DORC) tube injector was used to inject the tissue into the anterior chamber (AC). The tissue was opened and positioned using a tapping method. SF_6_ 20 % was injected for a full AC gas fill for 8 min, followed by reduction of the AC gas fill to 80 %. Her postoperative course was uncomplicated, with DMEK graft fully attached 360 degrees and improvement of uncorrected VA to 20/20 OS over the next three months. IOL position was unchanged compared to its preoperative position. Cornea remained clear (Fig. 3).

## Discussion and conclusions

With its advantages of minimal invasiveness and excellent postoperative visual outcomes, DMEK surgery has been performed with increasing frequency compared to PK and DSAEK for corneal decompensation for a variety of etiologies [1, 8]. PK would have addressed RK scars as well as endothelial dysfunction, but the patient would have to potentially endure the longest postoperative recovery course. DSAEK and ultra-thin DSAEK have previously been shown to induce a postoperative hyperopic shift and worse visual acuity outcomes than DMEK [9–11]. At our institution, DSAEK is often considered in aphakia, eyes with a glaucoma drainage device, or prior filtering surgery, and eyes in which the view of the AC is considered inadequate for manipulation of a DMEK graft. Since our patient did not meet any of these criteria and was most interested in optimizing postoperative visual outcome, DMEK was selected. However, DMEK may prove difficult if the AC or cornea are altered in any way.

**Fig. 4:**
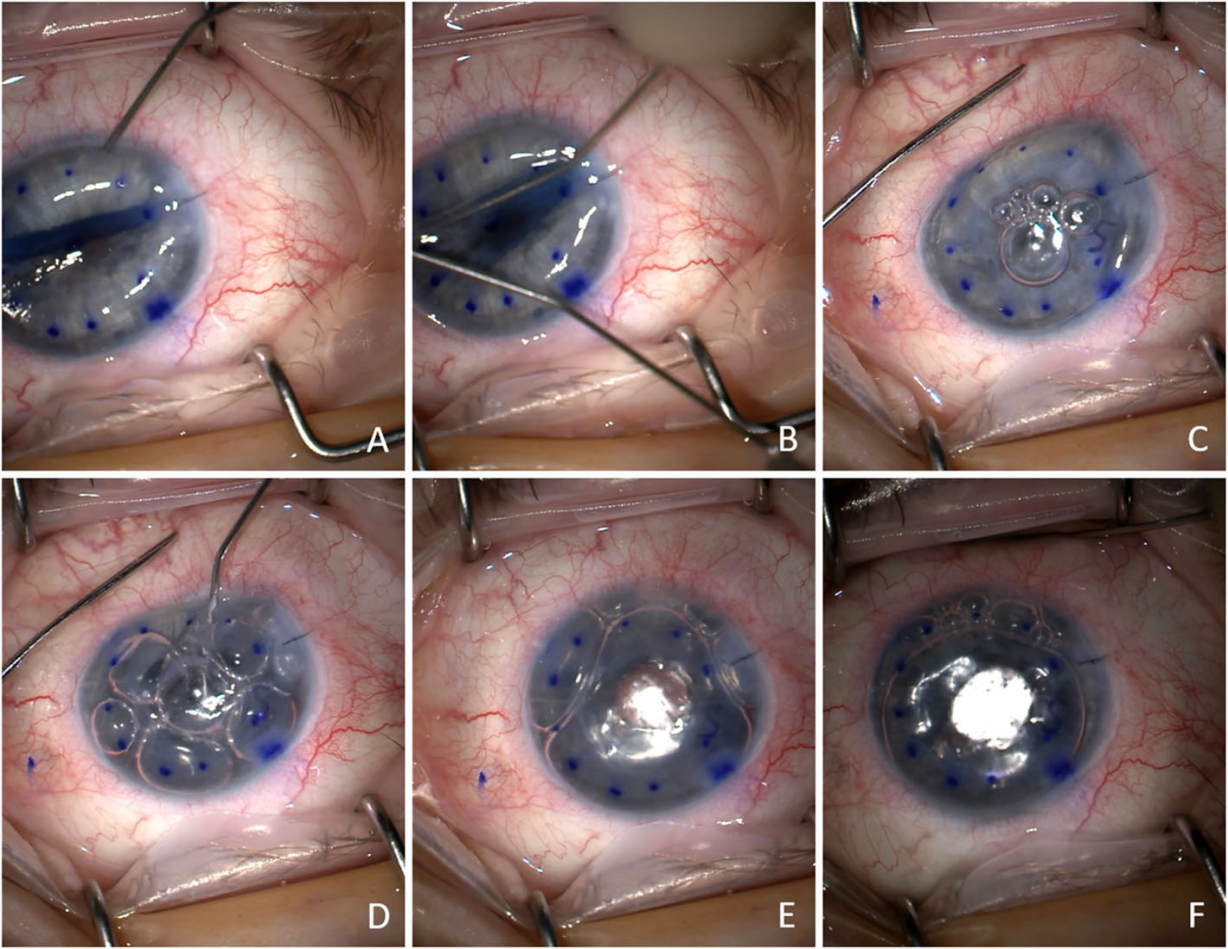
Intraoperative images demonstrating surgical challenges in DMEK in the context of RK and IF-IOL and techniques to overcome them. (**A**) Inversion of the corneal curvature during anterior chamber shallowing secondary to RK-associated structural weakening of the cornea. (**B**) Inversion of the corneal curvature during tapping secondary to RK-associated structural weakening of the cornea. (**C**) Use of a pilot bubble and external pressure on the sclera from a spatula to enable unfurling of the DMEK graft. (**D**) Use of external pressure on the sclera with a spatula during gas injection to ensure convexity of the graft and host cornea. (**E**) Full gas fill with DMEK graft unfurled and centered. (**F**) Final gas fill at the conclusion of the case

Potential challenges of DMEK in prior RK eyes include posterior corneal scarring (affecting the ability to strip Descemet membrane) and difficulty shallowing the AC, as patients are likely to be axial myopes (as was the patient reported here). Depending on the depth of the RK incision, rupture of an incision may occur during descemetorhexis. The RK incisions themselves weaken the structure of the cornea and can lead to frequent inversion of the corneal curvature during tapping and anterior chamber shallowing (Fig. 4 A-B). This leaves a moderate to shallow central AC, but a deep paracentral and peripheral AC, making the elimination of peripheral scrolling of the DMEK graft more difficult. Despite these challenges, several cases have been reported of DMEK under RK [5, 6]. In our patient, we used a pilot bubble (Fig. 4 C) to facilitate graft manipulation through partial fixation of the central graft during unfurling of the peripheral graft. Sweeping of the pilot bubble through the peripheral AC aided in shallowing the deep peripheral AC that resulted from intermittent inversion of the corneal curvature. This made it possible to eliminate peripheral curling of the DMEK graft. We also used external pressure on the sclera in conjunction with a pilot bubble to aid in maintaining convexity of the host cornea and graft (Fig. 4 C-D) during unfurling and gas injection. An additional movie file shows key challenges of the case and surgical modifications (see Additional file 1). Though it was not required in this case, posterior segment air infusion could be considered to improve the stability of the AC in post-vitrectomy eyes such as the one described here.



**Additional file 1.**



Further challenges may be present with potentially unstable IOLs such as IF-IOLs [12]. The goal of iris fixation was to decrease negative dysphotopsias by reducing the posterior chamber depth [13]. This method minimizes the distance between the IOL optic and the pupil, making it less likely that the edge of the IOL optic would cast a shadow on the peripheral retina. Prior groups have shown the feasibility of performing DMEK in patients with AC IOLs with good outcomes [14, 15]. Given the stability of the patient’s existing IF-IOL, we did not feel it necessary to explant it prior to proceeding with endothelial keratoplasty.

A critical step in DMEK surgery is final fixation of the graft under a gas bubble, which poses a risk of IOL dislocation into the vitreous chamber, especially in previously vitrectomized eyes [7]. Due to iris fixation of the IOL, the pupil diameter could be larger than ideal. Rock, et al. describe one case of successful DMEK in a patient with previous IF-IOL. However, it is important to note that an Artisan lens was used in their case, which is not available for aphakia in the United States. The Artisan IOL is fixated to the iris with claws which are enclavated onto the iris, not sutured [16]. The monofilament haptics of our patient’s 3-piece IOL were sutured to the iris at two locations, allowing for tilt of the IOL with tapping to manipulate the DMEK graft. This allows for the possibility of loss of the DMEK tissue into the posterior segment. This risk can be slightly mitigated by utilizing a lens with a large optic, such as the 6.0mm optic used in our patient. IOLs sutured to the iris also allow for movement of air or gas into the posterior segment, which can make placement of an adequate anterior chamber gas bubble more difficult. Use of external posterior segment pressure can temporarily shallow the anterior chamber to aid with unfurling of the graft.

Of note, our patient’s preoperative astigmatism was + 1.25 diopters, which decreased to + 0.75 diopters at the three-month postoperative visit. Though generally endorsed as a refractively near-neutral surgery, DMEK may potentially reshape the corneal surface, especially in eyes that have undergone anterior corneal manipulation [6, 17].

Here we have reported successful DMEK surgery in a patient with prior RK and an iris-sutured IOL (in addition to Fuchs dystrophy and prior anterior vitrectomy). There were no intraoperative complications, and uncorrected VA improved from 20/80 preoperatively to 20/20 postoperatively. Accordingly, while technically challenging, DMEK in eyes with RK and IF-IOL is possible and can offer effective visual rehabilitation.

## Data Availability

Data sharing is not applicable to this article as no datasets were generated or analyzed during the current study.

## Abbreviations

AC: Anterior chamber
BCVA: Best corrected visual acuity
DMEK: Descemet membrane endothelial keratoplasty
DORC: Dutch Ophthalmic Research Center
ERM: Epiretinal membrane
IF-IOL: Iris-fixated intraocular lens
IOL: Intraocular lens
OCT: Optical coherence tomography
OD: Right eye
OS: Left eye
OU: Both eyes
RK: Radial keratotomy
SF_6_: Sulfur hexafluoride
VA: Visual acuity
